# Artificial intelligence in total and unicompartmental knee arthroplasty

**DOI:** 10.1186/s12891-024-07516-9

**Published:** 2024-07-22

**Authors:** Umile Giuseppe Longo, Sergio De Salvatore, Federica Valente, Mariajose Villa Corta, Bruno Violante, Kristian Samuelsson

**Affiliations:** 1 Fondazione Policlinico Universitario Campus Bio-Medico, Via Alvaro del Portillo, Rome, 200 - 00128 Italy; 2 Department of Medicine and Surgery, Research Unit of Orthopaedic and Trauma Surgery, Università Campus Bio-Medico Di Roma, Via Alvaro del Portillo, Rome, 21 - 00128 Italy; 3 IRCCS Ospedale Pediatrico Bambino Gesù, Rome, Italy; 4 Orthopedic Unit, Department of Surgery, Bambino Gesù Children’s Hospital, Rome, Italy; 5 Orthopaedic Department, Clinical Institute Sant’Ambrogio, IRCCS - Galeazzi, Milan, Italy

**Keywords:** AI, Artificial intelligence, Machine Learning, Orthopaedics, Joint replacement, Knee replacement

## Abstract

The application of Artificial intelligence (AI) and machine learning (ML) tools in total (TKA) and unicompartmental knee arthroplasty (UKA) emerges with the potential to improve patient-centered decision-making and outcome prediction in orthopedics, as ML algorithms can generate patient-specific risk models. This review aims to evaluate the potential of the application of AI/ML models in the prediction of TKA outcomes and the identification of populations at risk.

An extensive search in the following databases: MEDLINE, Scopus, Cinahl, Google Scholar, and EMBASE was conducted using the PIOS approach to formulate the research question. The PRISMA guideline was used for reporting the evidence of the data extracted. A modified eight-item MINORS checklist was employed for the quality assessment. The databases were screened from the inception to June 2022.

Forty-four out of the 542 initially selected articles were eligible for the data analysis; 5 further articles were identified and added to the review from the PUBMED database, for a total of 49 articles included. A total of 2,595,780 patients were identified, with an overall average age of the patients of 70.2 years ± 7.9 years old. The five most common AI/ML models identified in the selected articles were: RF, in 38.77% of studies; GBM, in 36.73% of studies; ANN in 34.7% of articles; LR, in 32.65%; SVM in 26.53% of articles.

This systematic review evaluated the possible uses of AI/ML models in TKA, highlighting their potential to lead to more accurate predictions, less time-consuming data processing, and improved decision-making, all while minimizing user input bias to provide risk-based patient-specific care.

## Introduction

Artificial intelligence (AI) and Machine learning (ML) tools in knee arthroplasty (KA) have the potential to improve patient-centered decision-making and outcome prediction in orthopedics. The application of ML in KA has been useful for predicting implant size, reconstructing data, and assisting with component positioning and alignment. ML implementation enhances surgical precision and can help predict parameters such as length of hospitalization, healthcare costs, and discharge disposition [1–3]. 

Additionally, ML algorithms have been proven, in more recent studies, to be useful when selecting the right drugs to treat prosthetic joint infection (PJI) to have a more patient specific approach to medicine; this was possible due to the development of a Random Forest (RF) model able to take notice of several risk variables, such as patients’ characteristics and comorbidities and using the, for the selection [4]. In data science theory, the quantity and quality of input parameters are crucial; therefore, the previously mentioned variables, if not selected by relevance to the topic of each study, although beneficial in theory, may hinder the full potential of ML algorithms for KA. This is because, analyzing all underlying relations between variables, with a large number of inputs the models may highlight irrelevant patterns, leading to a greater risk of overfitting: the algorithms perform significantly better with the training data in respect to the newly presented one [4, 5].

Moreover, patient satisfaction following primary KA is one of many outcome measures currently used to assess the efficacy of this procedure. Patients’ satisfaction is dependent on many factors such as age, gender, and the presence of comorbidities. Therefore, it is essential to understand the relationship between the variables underlying satisfaction to provide the best care and optimized postoperative care for KA patients. ML algorithms, capable of generating patient-specific risk models, appear to be very effective means to achieve this goal [6].

Overall, the application and use of ML and AI in orthopaedics are beneficial not only for the previously mentioned situations, but also for the identification of possible patients that are at high risk for severe walking limitations post-total knee arthroplasty [7], and the selection of high-risk patients who will require a blood transfusion after KA [8].

This review will focus on investigating which predictions are achievable by using AI and ML models in knee arthroplasty, identifying prerequisites for the effective use of this new approach. Moreover, the second aim is to highlight the latest findings of these technologies in predicting outcomes after KA.

## Materials and methods

### Study selection

The research question was defined by using a PIO approach: Population (P); Intervention (I); Comparison (C); Outcome (O). The objective of this systematic review was to investigate which outcomes can be assessed by using AI or ML models (I) in patients with knee osteoarthritis who underwent total (TKA) or unicompartmental (UKA) knee replacement (P). The following outcomes were considered: complications, costs, functional outcomes, revision rate, and postoperative satisfaction (O).

### Inclusion criteria

Only articles that evaluated AI/ML-based applications in clinical decision-making in knee arthroplasty were considered. Only original clinical studies written in English, Spanish, or Italian were screened.

### Exclusion criteria

Studies that did not evaluate AI/ML applications in KA. Studies with nonhuman subjects. Medical imaging analysis studies without explicit reference or application to KA. Inaccessible articles, conference abstracts, reviews, and editorials. No limits were placed on the level of evidence or publication date of the study.

### Search

Following the Preferred Reporting Items for Systematic Reviews and Meta-analysis (PRISMA) guidelines, a thorough literature search was conducted using the following string: ((((total) OR (unicompartmental or unicondylar)) AND (knee replacement)) AND (((artificial intelligence) OR (machine learning)) OR (algorithm))) AND ((((((((complications) OR ((blood) AND ((transfusion) OR (loss)))) OR (functional outcomes)) OR (revision)) OR (satisfaction)) OR (surgical technique)) OR ((length of stay) OR (hospitalization))) OR ((costs) OR (economic analysis))). The use of keywords was both combined and isolated. The following databases were used: MEDLINE (Medical Literature Analysis and Retrieval System Online), Scopus, Cinahl, Google Scholar, PUBMED, and EMBASE (Excerpta Medica Database). The reference lists of selected systematic reviews [2, 5] were searched for the selection of further studies. The authors (F.V. and M.V.C.) searched from June of 2022 to January 2024. The databases were screened from the inception to January 2024.

### Data collection process

**Fig. 1:**
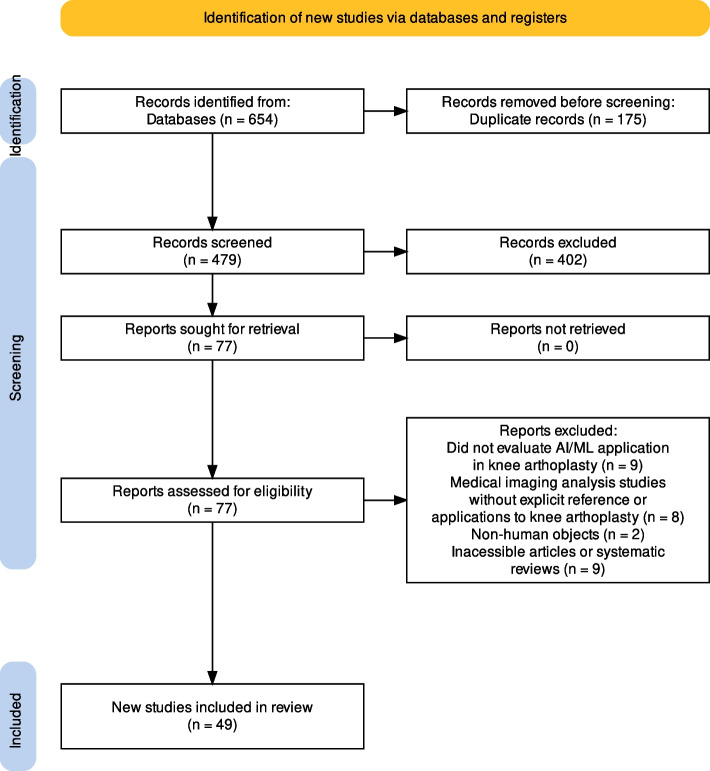
Prisma flowchart

Two independent reviewers (F.V. and M.V.C.) collected the data, and mutual approval resolved differences. A third reviewer (S.D.S) was consulted in case of any disagreement. Title and abstract screening were the first steps, followed by the full-text evaluation of the selected articles. The inclusion and exclusion of the reviewed studies were displayed in the PRISMA flowchart, seen in Fig. 1.

### Data items

A database was developed by collecting and categorizing the general study characteristics from the selected articles, which comprised: primary author, year of publication, study design, level of evidence, study duration, AI/ML methods, data source, input variables, output variables, sample size, average patient age, percentage of female patients, Area Under the Receiving Operating Characteristic Curve (AUC-ROC), accuracy, sensitivity, specificity.

### Risk of bias assessment

For the quality assessment, a modified eight-item Methodological Index for Non-Randomized Studies (MINORS) checklist was employed to evaluate the selected articles. The eight-item checklist included: disclosure, study aim, input feature, output feature, validation method, dataset distribution, performance metric, and AI model. Each item was scored using the following binary scale: 0 (not reported or unclear) and 1 (reported and adequate). The following criteria were used as a guide when assessing the quality of each publication: 

Disclosure: Scored 1 if clearly reported possible conflicts of interest, funding, or ethical considerations, scored 0 if not reported or unclear. Study aim: scored 1 if the research question and/or objective were clearly reported, scored 0 if unclear or not reported. Input feature: scored 1 if variables were clearly reported, scored 0 if unclear or not reported. Output feature: scored 1 if clearly reported, scored 0 if unclear or not reported. The validation method involves the evaluation of the AI/ML model’s performance by specific methods: scored 1 if the tools external validation, cross-validation, and/or bootstrapping were used and clearly reported, scored 0 if not reported nor used. Dataset distribution: scored 1 if the phases of training, testing, and validation for the AI/ML methods were clearly reported, scored 0 if unclear or not reported. Performance metric: scored 1 if the study clearly reported the metrics accuracy, sensitivity, specificity, and/or AUC-ROC for assessing the AI/ML model performance, scored 0 if unclear or not reported. AI model: scored 1 if clearly stated the specific AI/ML algorithm used by the study, scored 0 if not clearly stated.

Compared to the original MINORS checklist, this modified version, proposed by [9], provides a more accurate grading tool for studies focused on applying AI/ML methods in medical research and diagnostic studies within the medical field. Two independent reviewers (F.V. and M.V.C.) evaluated each publication individually.

## Results

### Study selection

The initial search identified 654 studies. After the duplicate removal, 479 studies were screened from which 402 articles were excluded after the title/abstract examination, resulting in 77 records for the full-text evaluation. After the full-text assessment, 49 studies were included in the data analysis (Fig. 1). Of these excluded articles, 9 studies did not evaluate AI/ML application in knee arthroplasty, 8 were medical imaging analysis studies without explicit reference or applications to knee arthroplasty, 2 used non-human objects, and 9 were inaccessible articles or systematic reviews.

### Study characteristics

A total of 2,595,780 patients were identified from 48 of the 49 studies included, with one study [10] not providing the sample size. Thirty-seven of the 49 studies stated the percentage of female patients, adding up to 1,435,218 female patients, which account for 55.29% of the total patients. The overall average age of the patients was 70.2 years ± 7.9 years old, with 33 out of 49 articles providing an average age of the study population. The study which had the highest number of patients was Hyer et al., 2020 [11] with 1,049,160 patients (40.41% of all the patients included in the studies). All the study characteristics are reported in Table 1.

**Table 1:** Study characteristics

Authors and year	Country	Study design	Level of evidence	Sample size	Average patient age	Percent female patients
Ben-Ari et al., 2017 [12]	USA	RCS	III	32,636	64.45 ± 9.41	5.6%
Bloomfield et al., 2019 [13]	Canada	RCS	III	68	67.5 ± 9.8	50%
Bonakdari et al., 2020 [10]	Canada	RCS	III	-	-	-
Bovonratwet et al., 2021 [14]	USA	RCS	III	319	63.1 ± 6.7	-
Chan et al., 2020 [15]	-	RCS	III	7	-	-
Crawford et al., 2023 [16]	USA	RCS	III	59	65	60.8%
Devana et al., 2021 [17]	USA	RCS	III	156,750	68.2 ± 9.2	61.4%
Farooq et al., 2020 [18]	USA	RCS	III	897	66.2 ± 8.9	72.6%
Farooq et al., 2021 [19]	USA	RCS	III	1,091	65.7 ± 9.3	67%
Fontana et al., 2019 [20]	USA	DS	III	6,480	66.9 ± 9.7	61%
Harris et al., 2019 [21]	-	DS	III	65,819	65.7	59.4%
Harris et al., 2021 [22]	USA	DS	III	637	-	-
Heisinger et al., 2020 [23]	-	RCS	III	165	64.5 ± 8.4	60%
Hinterwimmer et al., 2022 [5]	Germany	-	IV	864	66.5 ± 11.9	56.5%
Hsieh et al., 2020 [24]	Taiwan	RCS	III	26	69.15 ± 6.71	87.77%
Huang et al., 2018 [25]	-	RCS	III	15,187	62 ± 14.9	66%
Huber et al., 2019 [26]	UK	RCS	III	34,110	60–79	56.44%
Hyer et al., 2020 [11]	USA	RCS	III	1,049,160	73	55.8%
Jamshidi et al., 2021 [27]	Canada	RCS	III	1107	-	-
Jayakumar et al., 2021 [28]	-	RCS	III	69	-	67% (c: 62%)
Jo et al., 2020 [8]	-	RCS	III	1,686	74.5 ± 6.8	87.8%
Johannesdottir et al., 2022 [29]	Denmark	RCS	III	4,448	68	59%
Jones et al., 2016 [30]	UK	RCS	III	145	65 (UKA); 68 (TKA); 32 (Healthy controls)	-
Karnuta et al., 2019 [31]	-	RCS	III	159,726	-	66.32%
Katakam et al., 2020 [32]	USA	RCS	III	12,542	67 ± 7	60.3%
Katakam et al., 2022 [33]	USA	RCS	III	744	68	60.8%
Ko et al., 2022 [34]	-	PCS	II	5,757	71.2 ± 6.9	88%
Kunze et al., 2020 [6]	USA	RCS	III	430	66.2	68.8%
Kunze et al., 2021 [35]	-	RCS	III	17,283	66.3 ± 9.4	57.1%
Leung et al., 2020 [36]	USA	RCS	III	728	64 ± 8	61%
Li et al., 2022 [37]	Singapore	RCS	III	1,826	67.86 ± 8.13	77.98%
Mohammed et al., 2022 [38]	-	RCS	III	636,062	-	62.33%
Navarro et al., 2018 [39]	-	RCS	III	141,446	-	-
Pua et al., 2020 [7]	-	PCS	II	4,026	67.9 ± 7.5	75%
Rajamohan et al., 2023 [40]	USA	RCS	III	706	-	-
Ramazanian et al., 2022 [41]	USA	CS	II	4901	66 ± 10.4	55%
Ramkumar et al., 2019 [42]	-	RCS	III	171,025	73.53	64%
Ramkumar et al., 2019 [43]	-	CPS	IV	25	-	-
Rexwinkle et al., 2018 [44]	USA	-	-	6	63	33.3%
Sachau et al., 2022 [45]	Denmark	PS	IV	100	62.9 ± 9.6	66%
Sagheb et al., 2021 [46]	-	RCS	III	19,954	-	-
Shohat et al., 2020 [4]	UK	MCRS	III	609	70	54.2%
Tolpadi et al., 2020 [47]	USA	RCS	III	719	-	-
Tsai et al., 2023 [48]	Taiwan	RCS	III	3495	73	77%
Verstraete et al., 2020 [49]	USA	RCS	III	479	-	-
Wei et al., 2021 [50]	-	RCS	III	28,742	-	54.1%—66.1%
Yeo et al., 2023 [51]	USA	RCS	III	10,021	74.2 ± 22.7	60.16%
Yi et al., 2020 [52]	USA	RCS	III	690	-	-
Zhang et al., 2022 [53]	USA	PCS	II	2,008	66.3 ± 8.2	70.7%

*CPS* Cohort pilot study, *CS* Comparative study, *DS* Diagnostic study, *MCRS* Multi center retrospective study, *PCS* Prospective cohort study, *PS* Pilot study, *RCS* Retrospective cohort study

The five most common AI/ML models used were: RF, used in 19 articles; Gradient Boosting Machine (GBM), used in 18 articles (including less generalized versions such as Extreme Gradient Boosting (XGBoost) and Stochastic Gradient Boosting (SGB)); Artificial Neural Network (ANN) used in 17 articles; Logistic regression (LR), used in 16 articles (together with less generalized versions such as Elastic-net penalized logistic regression (EPLR)); and Support Vector Machine (SVM) used in 13 articles.

Regarding the variables reported, the most common input variables were: Age [38, 41, 45, 47, 49, 50, 52] (44 articles), Sex (33 articles), Comorbidities (29 articles), BMI (27 articles), Race/ ethnicity (26 articles), ASA classification (10 articles). The most common output variables provided by the studies were: post-surgical complications (11 articles), Probability of TKA (7 articles), and length of stay (4 articles).

This review included studies with level of evidence II-IV. Level of evidence II studies consist of Randomized controlled trials (RCTs) and are considered one of the strongest study designs, second only to reviews and meta-analysis which are considered as level of evidence I; Level of evidence III studies are composed of non-randomized controlled trials; the last category of evidence included in the review is Level IV: Case–control studies assessing associations between exposure and outcome.

The following level of evidence was included in the selected articles: 37 level III retrospective cohort studies [6, 8, 10–19, 23–37, 40, 48, 51]; three level III diagnostic studies [20–22, 54]; three level II prospective cohort studies [4, 39, 53]; one level II comparative studies [46]; three level IV cohort pilot studies [42, 44, 55], one level III multi-center retrospective study [47]. One study [43] did not present the level of evidence. All the characteristics are reported in Tables 1, 2 and 3.

**Table 2:** AI/ML methods

Authors and year	Data sources	AI/ML methods	AUC	Accuracy	Sensitivity	Specificity	
Ben-Ari et al., 2017 [12]	National VA databases	NLPM	-	0.94	-	-	
Bloomfield et al., 2019 [13]	Orthopedic database	-	-	-	-	-	
Bonakdari et al., 2020 [10]	Hawley et al. study (2018)	non-linear-GMHD	-	-	-	-	
Bovonratwet et al., 2021 [14]	-	NLPM	-	-	-	-	
Chan et al., 2020 [15]	-	RF	-	MTBCC: 0.78 MCBC: 0.82 CBC: 0.78	-	-	
		Multilayer perceptron (neural network)	-	MTBCC: 0.76 MCBC: 0.82 CBC: 0.82	-	-	
	-	SVM*	-	MTBCC: 0.76 MCBC: 0.80 CBC: 0.82	-	-	
Crawford et al., 2023 [16]	-	SGB	0.83	-	-	-	
	-	RF	0.83	-	-	-	
	-	SVM	0.79	-	-	-	
	-	ANN	0.78	-	-	-	
	-	EPLR	0.78	-	-	-	
Devana et al., 2021 [17]	OSHPD database	LR	0.629 ± 0.01	-	-	-	
		XGBoost	0.601 ± 0.03	-	-	-	
		GBM	0.662 ± 0.04	-	-	-	
		AdaBoost	0.657 ± 0.03	-	-	-	
		RF	0.545 ± 0.02	-	-	-	
		AP	0.679 ± 0.04	-	-	-	
Farooq et al., 2020 [18]	Local database	GBM	0.81	-	0.73	0.746	
Farooq et al., 2021 [19]	Local database	GBM	-	-	-	-	
Fontana et al., 2019 [20]	-	LASSO	0.60–0.89	-	-	-	
	-	RF	-	-	-	-	
	-	SVM	-	-	-	-	
Harris et al., 2019 [21]	NSQIP database	LASSO	-	Renal complication: 0.78; 30-day mortality: 0.73; Cardiac complications: 0.73	-		-
Harris et al., 2021 [22]	Local database (VA medical center)	LASSO	0.71	-	-	-	
		LASSO	0.72	-	-	-	
		GBM	0.72	-	-	-	
		QDA	0.72	-	-	-	
Hinterwimmer et al., 2022 [5]	EPRD, EndoCert	XGBoost	0.78	0.92	0.348	0.958	
Heisinger et al., 2020 [23]	OAI	ANN	-	-	-	-	
Hsieh et al., 2020 [24]	-	SVM	-	0.899	-	-	
	-	kNN	-	0.872	-	-	
	-	NB	-	0.863	-	-	
	-	DT	-	0.865	-	-	
	-	AdaBoost	-	0.908	-	-	
Huang et al.,2018 [25]	-	RF	0.85	-	-	-	
	-	LR	0.78	-	-	-	
Huber et al., 2019 [26]	NHS PRO data	XGBoost	0.87	0.78	0.82	0.77	
		RF	0.85	-	-	-	
		LR	0.86	0.77	-	-	
		NN	0.85	-	-	-	
		MSAENET	0.86	0.77	-	-	
		NB	0.75	-	0.99	-	
		KNN	0.62	-	-	-	
		LR	0.83	-	-	-	
Hyer et al., 2020 [11]	Medicare Inpatient, Outpatient SAFs	SHC	Perioperative morbidity: 0.868; 90-day readmission: 0.707; 30-day readmission: 0.717; postoperative super-use: 0.817; 30-day mortality: 0.834; 90-day mortality: 0.849	-	-		-
Jamshidi et al., 2021 [27]	OAI	Cox-PH model	0.87	-	-	-	
		DeepSurv/Nonlinear model	0.87	-	-	-	
		MTLR	-	-	-	-	
		MTLR	-	-	-	-	
		RF	-	-	-	-	
		SVM	0.87	-	-	-	
		SVM	-	-	-	-	
Jayakumar et al., 2021 [28]	US academic orthopedic practice	-	-	-	-	-	
Jo et al., 2020 [8]	ESSKA clinical data	GBM	0.842	-	0.898	0.748	
Johannesdottir et al., 2022 [29]	Lundbeck Centre for Fast-track hip and knee replacement Database, Danish National Patient Register	RF	0.71	0.75	0.44	0.82	
		SVM	0.71	0.73	0.52	0.78	
		NB	0.66	0.64	0.6	0.64	
Jones et al., 2016 [30]	Consultant surgeon, not specified	DT	-	-	-	-	
Karnuta et al., 2019 [31]	New York inpatient administrative database	MLP	0.791	-	-	-	
		DNN	0.813	-	-	-	
Katakam et al., 2020 [32]	-	SGB	0.76	-	-	-	
	-	RF	0.64	-	-	-	
	-	SVM	0.54	-	-	-	
	-	ANN	0.75	-	-	-	
	-	EPLR	0.76	-	-	-	
Katakam et al., 2022 [33]	-	SGB	0.74	-	-	-	
	-	RF	0.74	-	-	-	
	-	SVM	0.75	-	-	-	
	-	ANN	0.77	-	-	-	
	-	EPLR	0.76	-	-	-	
Ko et al., 2022 [34]	-	GBM	0.89	-	0.92	0.78	
Kunze et al., 2020 [6]	Electronic health records	SGB	0.79	-	-	-	
		RF	0.77	-	-	-	
		SVM	0.73	-	-	-	
		NN	0.66	-	-	-	
		EPLR	0.7	-	-	-	
Kunze et al., 2021 [35]	Community hospitals, Tertiary center	SGB	-	-	-	-	
		RF	-	-	-	-	
		SVM	-	-	-	-	
		GBM	-	-	-	-	
		LR	-	-	-	-	
Leung et al., 2020 [36]	OAI	DL-TL-MT	0.87	-	0.83	0.77	
		DL-TL	0.86	-	0.77	0.85	
		DL	0.84	-	0.70	0.85	
Li et al., 2022 [37]	-	XGBoost	0.738	-	-	-	
Mohammed et al., 2022 [38]	-	LR	DP: 0.685; C: 0.781; BT: 0.707	-	-	-	
	-	GBM	DP: 0.857; C: 0.871; BT: 0.797	-	-	-	
	-	RF	DP: 0.841; C: 0.847; BT: 0.783	-	-	-	
	-	ANN	DP: 0.848; C: 0.861; BT: 0.812	-	-	-	
Navarro et al., 2018 [39]	New York (SPARCS) administrative database	NB	0.782 (LOS); 0.738 (cost)	-	-	-	
Pua et al., 2020 [7]	Singapore General Hospital data	RF	0.74	-	-	-	
		XGBoost	0.755	-	-	-	
		SuperLearner	0.750–0.755	-	-	-	
		LR	0.751	-	-	-	
		LR + LASSO	0.750–0.755	-	-	-	
		LR + RIDGE	0.755–0.760	-	-	-	
Rajamohan et al., 2023 [40]	OAI, MOST	MLP	0.77	-	0.73	0.73	
		CNN	0.85–0.88	-	0.77–0.82	0.78–0.84	
		Ensemble models (MRI, MRI + Radiograph)	0.89–0.90	-	0.79–0.80	0.85–0.86	
Ramazanian et al., 2022 [41]	OAI, Institutional joint replacement registry	DL	-	-	-	-	
Ramkumar et al., 2019 [42]	NIS, OME	ANN	LOS: 0.832	LOS: 0.80	-	-	
			IC: 0.828	Inpatient Charges: 0.752	-	-	
			DD: 0.692	Discharge disposition: 0.644	-	-	
Ramkumar et al., 2019 [43]	RPM Application	ML-based Remote Patient Monitoring System	-	-	-	-	
Rexwinkle et al., 2018 [44]	-	ANN	-	-	-	-	
Sachau et al., 2022 [45]	-	RP	-	-	-	-	
	-	RF	-	-	-	-	
	-	KNN	-	-	-	-	
	-	NB	-	-	-	-	
	-	LR	-	-	-	-	
	-	LDA	-	-	-	-	
Sagheb et al., 2021 [46]	Mayo clinic database	NLPM	-	0.983	-	-	
Shohat et al., 2020 [4]	-	RF	0.74	-	-	-	
Tolpadi et al., 2020 [47]	OAI	LR	0.88	78.5 ± 0.134%	81.8 ± 0.643%	78.4 ± 0.138%	
		DNN	0.88	-	-	-	
Tsai et al., 2023 [48]	-	SORG-MLA	0.75	-	-	-	
Verstraete et al., 2020 [49]	-	RF	0.89	0.99	-	-	
	-	SVM	0.82	-	-	-	
	-	ANN	0.83	-	-	-	
Wei et al., 2021 [50]	NSQIP database	ANN	0.801	-	-	-	
		LR	0.796	-	-	-	
Yeo et al., 2023 [51]	-	ANN	0.84	-	-	-	
	-	SGB	0.79	-	-	-	
	-	SVM	0.78	-	-	-	
	-	RF	0.80	-	-	-	
	-	EPLR	0.80	-	-	-	
Yi et al., 2020 [52]	Publicly available websites: Radiopaedia, Google, Bing, National Institues of Health. Postoperative radiographs performed at Johns Hopkins University	DCNN	1	-	1	1	
Zhang et al., 2022 [53]	ESSKA clinical database	RF	0.89 (WOMAC)	-	0.832	0.766	
		XGBoost	SF-36 PCS: 0.77; SF-36 MCS: 0.95	SF-36 PCS: 0.752; SF-36 MCS: 0.956	SF-36 PCS: 0.671; SF-36 MCS: 0.849	-	
		SVM	SF-36 PCS: 0.76; SF-36 MCS: 0.95	SF-36 PCS: 0.764; SF-36 MCS: 0.931	SF-36 PCS: 0.638; SF-36 MCS: 0.868	-	
		LASSO	0.89 (WOMAC)	-	0.755	0.82	

*ANN* Artificial Neural Network, *AP* AutoPrognosis, *BT* Blood transfusion, *C* Complications, *CBC* Cartilage and bone classification, Cox-PH model, *DCNN* Deep Convolutional Neural Network, *DD* Discharge disposition, *DNN* DenseNet, *DL* Deep learning, *DP* Disposition of patient, *DT* Decision tree, *EPLR* Elastic-net Penalized Logistic Regression, *ESSKA* European Society of Sports Traumatology, Knee Surgery and Arthroscopy, *GBM* Gradient Boosting Machine, *IC* Impatient charges, *kNN* K-Nearest Neighbors, *LASSO* Least Absolute Shrinkage and Selection Operator, *LDA* Linear discriminant analysis, LR Logistic regression, *MCBC* Muscle, cartilage, bone classification, *MCS* Mental component summary, *MLP* Multilayer perceptron, *MT* Multitask, *MTBCC* Muscle, tendon, bone, cartilage classification, *MTLR* Multi-Task Logistic Regression, *MSAENET* Multi-Step Adaptive Elastic NETwork, *NB* Näive-Bayes, National VA databases: National Veteran’s affairs databases, *NHS* National health service, *NIS* National (Nationwide) Inpatient Sample database, *NN* Neural network, *NLPM* Natural Language Processing Method, *NSQIP* National Surgical Quality Improvement Program, *OAI* Osteoarthritis initiative, *OME* Orthopedic Minimal Data Set database, *OSHPD* Office of Statewide Health Planning and Development, *PCS* Physical component summary, *QDA* Quadrant Discriminant Analysis, *RF* Random forest, *RP* Recursive partitioning, *SAFs* Standard analytical files, *SGB* Stochastic Gradient Boosting, *SHC* Stochastic Hill Climbing, *SORG-MLA* Skeletal Oncology Research Group Machine Learning Algorithm, *SPARCS* State-wide Planning and Research Cooperative System, *SVM* Support Vector Machine, *TL* Transfer learning, *XGBoost* EXtreme Gradient Boosting

**Table 3:** Input and output variables

Authors and year	Input variables	Output variables (OV)
Ben-Ari et al., 2017 [12]	Age, BMI, Diabetes, Opioid use, Chronic Kidney disease	Alteration of opioid use in the risk of knee revision and knee manipulation in the 1st year after a primary TKA
Bloomfield et al., 2019 [13]	Age, BMI, UCLA Activity Score, SF-12 Mental, SF-12 Physical, WOMAC Pain, WOMAC Stiffness, WOMAC function, WOMAC Total, KSS symptoms, KSS satisfaction, KSS expectations, KSS Functional Activities, KSS Knee Objective Indicators, Knee Evaluation Function, Knee Evaluation Total Knee, Knee Evaluation Total	Functional performance (before & during) short term recovery
Bonakdari et al., 2020 [10]	-	-
Bovonratwet et al., 2021 [14]	Age, Gender, BMI, Insurance type, ASA classification, Number of allergies, Operative time (min), Anesthesia type, Length of stay, Non-homebound discharge, Received blood transfusion, Received Scopolamine patch, Straight catheterization, Inpatient peak pain intensity, Inpatient opioid intake	Identification of differences in postoperative outcomes, PROMs, satisfaction
Chan et al., 2020 [15]	-	Characterization of anatomical tissues
Crawford et al., 2023 [16]	Age, Sex, BMI, Race, Charlson comorbidity score (CCI), Diabetes, Chronic obstructive pulmonary disease (COPD), chronic kidney disease (CKD), Depression, Opioid use, Benzodiazepine use, Smoking status, Arthritis, Injection, Physical therapy, Assistive device	Operative intervention: yes or no
Devana et al., 2021 [17]	Age, Sex, Race, Ethnicity, Hospital volume range, Insurance, Comorbidities (CMS Clinical Condition), Total Complications	Major complications after primary TKA
Farooq et al., 2020 [18]	Age, BMI, LOS, FU, generation, sex, ASA, surgeon, type of implant, PCL addressed, Depression, Inflammatory cognition, pre-operative narcotic use, Lumbar spine pain/surgery/disease, Tourniquet	Patient satisfaction (identification of predictors)
Farooq et al., 2021 [19]	Age, BMI, Sex, ASA-PS, PCL status, Implant type, Navigation used, Fixation type, Tourniquet use, Inflammatory condition, Lumbar spina disease, Depression, Preoperative narcotic	Effect of sagittal component alignment in modern patient-reported outcomes
Fontana et al., 2019 [20]	Age, ASA score, Years of education, BMI, Operation time, LOS, Number of final procedures, diagnosis code	Achievement of MCIDs
Harris et al., 2019 [21]	Age, Gender, Race, BMI, ASA class, Medication and treatments, Preoperative conditions, Smoking status, Steroids medications, Open wound	Mortality & complications after TKA: 30-day mortality, 30-day cardiac complications, central nervous system-cardiovascular system complications, respiratory complications, surgical wound infection, return to the operating room, renal complications, venous thromboembolism
Harris et al., 2021 [22]	Age, BMI, sex, race/ethnicity, marital status, education, employment status, CHF, Valvular disease, Peripheral vascular disease, Hypertension, Neurological disorders, CP, DM, Hypothyroidism, Renal failure, Liver disease, solid tumor without metastasis, Rheumatoid arthritis, weight loss, fluid and electrolyte disorders, deficiency anemia, alcohol use disorder, drug use disorder, depression, AUDIT-C, PHQ, KOOS	Achievement of MCIDs in KOOS 1 year after TKA
Heisinger et al., 2020 [23]	Age, Sex, BMI, Ethnicity, Medication, Annual income, Education, Depression, Start of knee symptoms prior to baseline screening visit, TKA at which year	Performance of TKA based on factors in a four-year period prior to TKA surgery
Hinterwimmer et al., 2022 [5]	Age, Sex, Weight, Height, BMI, Diagnosis, Implant type, Side, Surgeon, Experience level of surgeon, Surgery type	Complications after TKA, duration of surgery
Hsieh et al., 2020 [24]	Time of TUG test subtasks	Subtask segmentation of TUG test for perioperative TKA
Huang et al.,2018 [25]	Age, Sex, BMI, Hypertension, Type 2 diabetes, ASA class, TXA use, Intraoperative blood loss, Drain use, Preoperative Hb	Predictors of ALBT
Huber et al., 2019 [26]	Age, Sex, Previous knee-replacement surgery, Disability, Mean preoperative VAS score, Mean preoperative Q score	Prediction of PROs
Hyer et al., 2020 [11]	Age, Sex, Race, Weight, Comorbidity	Prediction surgical 90-day morbidity, mortality, complications
Jamshidi et al., 2021 [27]	X-rays, Bone marrow Lesions in medial condyle, Hyaluronic acid Injections, Performance measure, Medical History, Knee symptoms	Risk and Time of TKA in OA knee
Jayakumar et al., 2021 [28]	Age, Sex, Ethnicity, Education, Work status, Social status, Insurance status, PHQ-depression, GAD-anxiety, Diabetes, Smoking status, Charlson Comorbidity Index, Duration of pain, BMA	Decision quality, Collaborative decision making, Patient satisfaction, KOOS JR score, Consultation time, TKA rate, Treatment accordance
Jo et al., 2020 [8]	Gender, age at surgery, ASA score, BMI, hypertension, autologous transfusion, preoperative Hb, preoperative creatinine, operation time, total blood loss, infused fluid decrease in Hb	Transfusion after TKA
Johannesdottir et al., 2022 [29]	Age, BMI, Sex, Smoking, Use of walking aid, Living alone, Joint operated on, Comorbidities	Prediction of LOS of > 2 days after fast-track total knee replacement
Jones et al., 2016 [30]	Age, BMI, Height, Ahlbäck Grade, Oxford Knee Score, Top speed recorded, Gait variables	Gait comparison between UKA and TKA patients
Karnuta et al., 2019 [31]	Risk of mortality, Severity of illness, Diagnosis code, Type of admission, Ethnicity, Race, Gender, Age, Primary diagnosis	Procedural cost for TKA
Katakam et al., 2020 [32]	Age, Sex, Race, Ethnicity, Laboratory Values, Medicaid, Medicare, preoperative opioid use, Marital status, Diabetes, Comorbidities, Preoperative medication	Prolonged postoperative opioid prescription
Katakam et al., 2022 [33]	Age, Sex, BMI, American Society of Anesthesiologist class, Medicaid, Medicare, Laboratory Values, Preoperative opioid use, Comorbidities	Improvement of KOOS to one-year postoperative score greater than or equal to the MCIDs
Ko et al., 2022 [34]	Sex, General anesthesia, Preoperative serum creatinine levels, ASA class, Use of RAASis, Use of tranexamic acid	Development of AKI effect of AKI on progression to End-Stage Renal Disease
Kunze et al., 2020 [6]	Age, Body mass index, Preoperative opioid use within 3 months prior to surgery, Smoking history, diabetes, Drug allergies, Number of comorbid conditions, Fibromyalgia/depression status, Prior ipsilateral knee procedure not including a TKA, Degree of knee flexion, PHRS, Preoperative KSS, Preoperative KSS-F scores	Dissatisfaction after TKA
Kunze et al., 2021 [35]	Age, BMI, Sex, Height, Weight, Femoral component implant size (mm), Tibial component implant size (mm)	TKA component size prediction (femoral and tibial)
Leung et al., 2020 [36]	Age, Height, Weight (kg), BMI (kg/m2), Ethnicity, KL grade	Prediction for risk of OA progression
Li et al., 2022 [37]	Age, Race, Gender, BMI, Hb level, Operation duration, Smoking, Diabetes mellitus, Cerebrovascular accident, Congestive heart failure, American Society of Anesthesiologist score, type of anesthesia, preoperative elevated creatine level	LOS after TKA
Mohammed et al., 2022 [38]	Age, Gender, Race, Admission month, Admission on a weekend, Health insurance, median household income for patient's zip code, Admission type, Health insurance, Patient geographical location, Ownership of hospital, location and teaching status of hospital, Comorbidities	Disposition of patients at discharge, post-surgical complications, blood transfusion
Navarro et al., 2018 [39]	age group, CCS, ethnicity, gender, patient disposition, type of admission, APR risk of mortality, APR severity of illness	LOS, Inpatient costs after first TKA
Pua et al., 2020 [7]	Age, weight, height, BMI, race, Contralateral knee pain, Hypertension, Dyslipidemia, Diabetes, Adult recon specialist, Caregiver available, Education level, Gait aids, Knee pain, Depression level, Anxiety level, Difficulty when climbing own stairs, Difficulty when kneeling and getting up, Knee flexion, Knee extension, SF-36 physical function, Walking limitation	Post-TKA walking limitation
Rajamohan et al., 2023 [40]	Age, Sex, Ethnicity, BMI, WOMAC score, KOOS QoL score, cartilage MOAK score, bone marrow edema lesion MOAK score	Prediction of TKA
Ramazanian et al., 2022 [41]	Age, Sex, Weight, Height, BMI, Arthritis etiology	HKA angle ± range comparison in knee OA patients
Ramkumar et al., 2019 [42]	Age, Gender, Ethnicity, Race, Type of Admission, APR Risk of mortality, APR Severity of illness, Number of associated conditions, comorbidity status, Weekend admission, hospital type, Income quartile, transferred from outside hospital, Bias Term	Prediction of LOS, inpatient charges, discharge disposition before primary TKA
Ramkumar et al., 2019 [43]	Mobility, Range of motion, PROMs, Opioid consumption, HEP compliance	Feasibility of RPM system for data interruption and patient acceptance
Rexwinkle et al., 2018 [44]	Age, Sex, BMI, OA, Biomarkers, Osteochondral samples	Prediction of key biomechanical properties of articular cartilage
Sachau et al., 2022 [45]	Age, Sex, BMI, Pain duration in the index knee, Pain intensity, Pain walking, Pain climbing stairs, KOOS, Pain DETECT questionnaire score, Neuropathic component, Pain Quality Assessment Scale	Assessment of sensitization in patients with chronic pain after TKA
Sagheb et al., 2021 [46]	-	Category of surgery (TKA, Unicompartmental knee arthroplasty, patellofemoral arthroplasty), Implant model (catalog numbers), Presence of patellar resurfacing, Constraint type, Laterality of surgery
Shohat et al., 2020 [4]	Age, Sex, BMI, Smoking, Alcohol, Comorbidities, Immunosuppression medications, History of infected prosthesis, Clinical findings, Laboratory findings, Operative factors, Organism profile, Timing	Failed treatment: yes or no
Tolpadi et al., 2020 [47]	Age, BMI, Education, Ethnicity, Income, NSAID usage, Analgesics usage, Systolic BP, Considering TKA, PASE, KOOD QOL, KOOS pain, WOMAC pain, WOMAC disability, Comorbidity score, Injections to treat arthritis in previous 6 months, Seen physician for arthritis in previous year, Knee valgus negative alignment, Isometric leg strength, Back pain in previous 30 days, Difficulty squatting in previous 7 days, Difficulty kneeling in previous 7 days, Baseline frequent knee pain status, Previous knee injury that limited walking, o-10 global rating assessing effect of knee pain, SF-12 physical component score, SF-12 mental component score	Probability of TKA within 5 years
Tsai et al., 2023 [48]	Age, Sex, Marital status, Ethnicity, Preoperative laboratory values, Median household income, educational level, neighborhood unemployment rate, Preoperative medications, Comorbidities	Prolonged postoperative opioid use
Verstraete et al., 2020 [49]	Surgical decisions, Medial load at 10°, Lateral load at 10°, Medial load at 90°, Lateral load at 90°, Varys/Valgus deformity pre-op, Max extension deformity pre-op, Varys/Valgus during trialing, Max extensions during trialing	Surgical corrections based on patient-specific intra-operative assessments
Wei et al., 2021 [50]	Gender, Race, Diabetes, ASA grade, Dyspnea status, Functional status, Anemia WHO class, Anesthesia type	Prediction same-day discharge in patients undergoing TKA
Yeo et al., 2023 [51]	Age, Gender, BMI, Laterality, ASA score, Charlson Comorbidity Index, Insurance status, Ethnicity, Preoperative medications, Follow-up time, Comorbidities, Surgical variables	Prediction of surgical site infection following TKA
Yi et al., 2020 [52]	Anteroposterior knee radiographs with equal proportions of native knees TKA and UKA; AP knee radiographs with equal proportions of two TKA models	Identification of presence or absence of a TKA, differentiation between two different primary TKA models, Classification of TKA vs UKA
Zhang et al., 2022 [53]	Age, sex, race, BMI, Payment class, Diabetes, Hypertension, Ischemic Heart Disease, Stroke, Cancer, Respiratory disease, Preop Pain Score, Surgeon Preop PCS, Preop MCS, Preop WOMAC	MCIDs attainment at 2 years after TKA

*AKI* Acute Kidney Infection, *ALBT* Allogenic blood transfusion, *APR* All patient refined, *ASA-PS* American society of anesthesiologists physical status, *ASA* American Society of Anesthesiologists, *BMI* Body mass index, *BP* Blood pressure, *CCS* Charlson comorbidity score, *CMS* Centers for Medicare & Medicaids Services, *Hb* Heamoglobin, *HEP* Home exercise program, *UCLA* University of California Los Angeles, *KL* Kellgren-Lawrence score, *KOOS* Knee injury and osteoarthritis outcome score, *KSS-F* Knee society score function, *KSS* Knee society score, *LOS* Length of stay, *MCIDs* Minimally clinically important difference, *MCS* Mechanical circulatory support, *NSAID usage* Non-steroidal anti-inflammatory drugs, *OA* Osteoarthritis, *PASE* Physical activity scale for the Elderly, *PCL* Posterior cruciate ligament, *PCS* Previous cardiac surgery, *KSS* Knee society score, *KSS-F* KSS-Function (KSS-F) scores, *PHRS* Preoperative patient-reported health state, *PROMs* Patient reported outcome measures, *PROs* Prediction of patient-reported outcomes, *QOL* Quality of life, *RPM* Remote patient monitoring, *RPM* Remote patient monitoring, *SF-36* Short form, *TKA* Total knee arthroplasty, *TUG* Time up and go, *UKA* Unicompartmental knee arthroplasty, *VAS* Visual analog scale, *WHO* World health organization, *WOMAC* Western Ontario and McMaster Universities

### AI and ML methods

The following section reports the AI and ML methods identified in the reviewed articles. Each section includes the number of articles that used each AI or ML method, its corresponding AUC value, and the evaluated output variable. Table 4 classifies each article regarding the output variable studies and presents the highest AUC score for the respective article.

**Table 4:** Output variables

Output	Authors and year	AI/ML	AUC
**Length of Hospital Stay (LOS)**	Johannesdottir et al., 2022 [29]	RF, SVM	0.71
	Li et al., 2022 [37]	ANN, XGBoost	0.738
	Navarro et al., 2018 [39]	NB	0.782
	Ramkumar et al., 2019 [42]	ANN	0.832
**Complications**	Devana et al., 2021 [17]	AP	0.679 ± 0.04
	Harris et al., 2019 [21]	LASSO	-
	Hinterwimmer et al., 2022 [5]	XGBoost	0.78
	Hyer et al., 2020 [11]	SHC	Perioperative morbidity: 0.868; 90-day readmission: 0.707; 30-day readmission: 0.717; postoperative super-use: 0.817; 30-day mortality: 0.834; 90-day mortality: 0.849
	Ko et al., 2022 [34]	GBM	0.89
	Mohammed et al., 2022 [38]	GBM	0.871
	Yeo et al., 2023 [51]	ANN, EPLR, RF, SGB, SVM	0.78–0.84
**Blood transfusion**	Huang et al.,2018 [25]	RF, SVM	0.85
	Jo et al., 2020 [8]	GBM	0.842
	Mohammed et al., 2022 [38]	ANN	0.812
**Inpatient cost**	Navarro et al., 2018 [39]	NB	0.738
	Ramkumar et al., 2019 [42]	ANN	0.828
**Cost Prediction**	Karnuta et al., 2019 [31]	DenseNet	0.813
**Future Clinical Intervention**	Ben-Ari et al., 2017 [12]	NLPM	-
	Crawford et al., 2023 [16]	ANN, EPLR, RF, SGB, SVM	0.78–0.83
	Heisinger et al., 2020 [23]	ANN	-
	Jamshidi et al., 2021 [27]	Cox-PH, DeepSurv, SVM	0.87
	Leung et al., 2020 [36]	DL-TL-MT	0.87
	Rajamohan et al., 2023 [40]	MLP, CNN, Ensemble model	0.77–0.90
	Tolpadi et al., 2020 [47]	LR, DNN	0.88
**Clinical outcomes**	Farooq et al., 2021 [19]	TreeNet GBM	-
	Katakam et al., 2020 [32]	EPLR, SGB	0.76
	Sachau et al., 2022 [45]	RF	-
	Shohat et al., 2020 [4]	RF	0.74
	Tsai et al., 2023 [48]	SORG-MLA	0.75
	Wei et al., 2021 [50]	ANN	0.801
	Mohammed et al., 2022 [38]	GBM	0.857
*Patient Satisfaction*	Farooq et al., 2020 [18]	TreeNet GBM	0.81
	Kunze et al., 2020 [6]	SGB	0.79
*MCIDs, KOOS, PROs*	Fontana et al., 2019 [20]	LASSO	0.60–0.89
	Harris et al., 2021 [22]	LASSO, GBM, QDA	0.72
	Katakam et al., 2022 [33]	ANN	0.77
	Jayakumar et al., 2021 [28]	-	-
	Zhang et al., 2022 [53]	RF, LASSO	0.89 (WOMAC)
		XGB, SVM	0.95 (MCS)
	Huber et al., 2019 [26]	XGBoost	0.87
**Functional outcomes**	Bloomfield et al., 2019 [13]	-	-
	Hsieh et al., 2020 [24]	AdaBoost	-
	Pua et al., 2020 [7]	LR + ridge	0.755–0.76
**Surgical technique/outcomes**	Chan et al., 2020 [15]	RF	-
	Hinterwimmer et al., 2022 [5]	XGBoost	0.78
	Jones et al., 2016 [30]	DT	-
	Sagheb et al., 2021 [46]	NLPM	-
	Verstraete et al., 2020 [49]	RF	0.89
	Yi et al., 2020 [52]	DCNN	1
**Technical outcomes / biomechanical properties**	Kunze et al., 2021 [35]	XGBoost, SGB, EPLR, SVM, RF,	-
	Ramazanian et al., 2022 [41]	DL algorithm	-
	Ramkumar et al., 2019 [43]	ML-based Remote Patient Monitoring System	-
	Rexwinkle et al., 2018 [44]	ANN	-

*AKI* Acute Kidney Infection, *ALBT* Allogenic Blood Transfusion, *DL-TL-MT* Deep Learning – Transfer Learning – Multitask, *ANN* Artificial Neural Network, *AP* AutoPrognosis, *CoxPH* Cox proportional hazards, *DCNN* Deep Convolutional Neural Network, *DenseNet* Densely Connected Convolutional Network, *DS* DeepSurv, *DT* Decision tree, *EPLR* Elastic-net Penalized Logistic Regression, *EPLR* Elastic-net Penalized Logistic Regression, *GBM* Gradient Boosting Machine, *HKA* Hip-knee-angle, *IC* Inpatient costs, *KOOS JR* Knee injury and Osteoarthritis Outcome Score for Joint Replacement, *KOOS* Knee Injury and Osteoarthritis Outcome Score, *LASSO* Least Absolute Shrinkage and Selection Operator, *LOS* Length of stay, *LR* Logistic Regression, *MCIDs* minimally clinically important differences, *MLP* Multilayer perceptron, *NB* Näive-Bayes, *NLPM* Natural Language Processing Method, *OA* Osteoarthritis, *PROs* Patient-reported outcomes, *QDA* Quadrant Discriminant Analysis, *RF* Random Forest, *RPM* Remote patient monitoring, *SGB* Stochastic Gradient Boosting, *SORG-MLA* Skeletal Oncology Research Group Machine Learning Algorithm, *SVM* Support Vector Machines, *TKA* Total Knee Arthroplasty, *TUG* Time Up and Go test, *XGBoost* EXtreme Gradient Boosting

### Random forest

RF is a decision trees-based algorithm introduced in the 2000s and capable of handling a variety of data types; its implementation in many medical fields is sustained by its high performance with large datasets and its ability to integrate both clinical and imaging data to achieve more accurate predictions compared to older models such as LR. This ML method operates by constructing and averaging a multitude of decision tress, a simpler ML method, with each of the tress randomly analyzing selected subset variations of the original data, the model is capable to analyze large and complex subset of data, resulting in a more resistant model to overfitting, while also adding diversity in the analysis. It was the most common AI method, applied in 38.77% of the reviewed articles. Mainly it was used to evaluate outcomes, one of them being a technical outcome: TKA component size prediction (femoral and tibial) [35]. Eight publications implemented RF for the evaluation of clinical outcomes, some of them being: achievement of Minimal Clinically Important Differences (MCIDs), prediction of Patient Reported Outcomes (PROs), prolonged postoperative opioid prescription, improvement of Knee injury and Osteoarthritis Outcome Score (KOOS) to one-year, dissatisfaction, assessment of sensitization in patients with chronic pain after TKA, etc. [4, 6, 20, 26, 32, 33, 45, 53]. Only one article evaluated the post-walking limitation with RF, under the functional outcome category [7, 56].

RF was also utilized to analyze the surgical technique by two articles [15, 49], which considered the following outputs respectively: characterization of anatomical tissues and surgical corrections, the latter presenting the highest AUC (0.89) for this ML method. Postoperative length of stay (LOS) was predicted using RF only by one article [57], which presented an AUC of 0.71.

Another application of RF was regarding possible complications such as major complications after primary TKA, blood transfusion, surgical site infection, and disposition of patients at discharge [15, 25, 38]. Lastly, two reviewed articles implemented RF for predicting TKA risk depending on knee OA, evaluating both risk and time [16, 27].

### Gradient boosting machine

The ML model GBM gained popularity in the 2000s due to the model’s high predictive accuracy even in settings with mixed data types and missing values. GBM works by building decision tress sequentially, rather than in parallel like RF, with each of the tress correcting the predicting errors made by the previous ones. This results in the model being able to analyse complex relationships in data and producing an accurate prediction, even if lacking the randomized selection or diversity of the RF model. It can be used for both classification and regression due to its ability to produce new decision trees by correcting the errors of the previous predictions, gaining more accuracy than popularly used models such as SVM.

It was used by 18 studies, one employing it to predict TKA component size [35]. The highest AUC value was applied by an article that evaluated the development of acute kidney infection (AKI) after TKA, AUC: 0.89 [34]. Other studies that evaluated complications with GBM comprised the following outputs: major complications after primary TKA, blood transfusion after TKA, surgical site infection, and disposition of patients at discharge [8, 17, 38]. One study used GBM for the prediction of LOS after TKA [37], a different study employed this method to evaluate functional outcome: post-TKA walking limitations [7].

In addition, GB was used by 7 articles to evaluate clinical outcomes: prediction of patient satisfaction, achievement of MCIDs in KOOS 1 year after TKA, prediction of PROs, extended prescription of postoperative opioids, MCIDs attainment 2 years after TKA [6, 18, 19, 22, 26, 32, 33, 53]. Only one study evaluated the use of SGB to predict the risk of TKA in comparison to other ML models, resulting in the highest performance together with RF among the algorithms observed, with an AUC: 0.83 [16].

### Artificial Neural Network (ANN) /Multilayer perceptron

Although it originated in the 1940s, the ANN model gained prominence in the 2010s due to the application of deep learning in modeling complex relationships, making it suitable for a wide range of applications. ANN is a computational algorithm consisting of interconnected nodes organized in sequential layers, each analyzing the data to pass the prediction to the following one, mimicking the functioning of human neural network. This model was applied by 17 studies, one of them being for the prediction of LOS, inpatient charges, and discharge disposition before primary TKA [43]. Five articles analyzed clinical outcomes, the one having the highest AUC for this method (0.86) was regarding the prediction of PROs [26]; other outputs under this category were: prolonged postoperative opioid prescription, dissatisfaction after TKA, prediction of same-day discharge in patients undergoing TKA [6, 32, 33, 50]. One article applied ANN for TKA component size prediction (femoral and tibial) [44], and another study applied it for procedural cost prediction for TKA [31, 58].

Regarding complications, ANN was applied to evaluate the disposition of patients at discharge, post-surgical complications such as surgical site infection, and blood transfusion [38]. Additionally, two articles used this ML method to characterize tissues and surgical corrections based on patient-specific intra-operative assessment [15, 49]. Another application of ANN, by four other articles, was related to future clinical intervention outputs: effect of opioid use in risk of knee revision and manipulation in the first year after primary TKA [59]; identification of influential factors before surgery, and prediction of the risk of TKA surgery [23, 60].

### Logistic regression

LR is a simply interpretable model for binary classification developed in the early twentieth century; being one of the oldest predictive models, its role is well established in the medical setting to estimate the probability of occurrence of different events. Although, it is to be considered that the advent of newer algorithms able to form wider and more complex associations between inputs and outputs causes this model to be more frequently relegated to a comparator role. The algorithm was used by 16 out of 49 articles. Four articles evaluated complications, which comprised the following outputs: disposition of patient at discharge, predictors of Allogenic Blood Transfusion (ALBT), and post-surgical complications [17, 25, 38]. The future clinical intervention was studied by three articles, specifically regarding the risk and time for a TKA in a patient presenting knee OA [27]. One article used this machine learning method for TKA component size prediction [35], and a different publication used it to evaluate post-TKA walking limitations, a type of functional outcome [7].

Regarding clinical outcome, LR was applied by 7 articles to study: achievement of MCIDs in KOOS 1 year after TKA, extended opioid prescription post-surgery, dissatisfaction after TKA, assessment of sensitization in patients with chronic pain after TKA, prediction of same-day discharge in patients undergoing TKA, and prediction of PROs [6, 22, 26, 32, 33, 45, 50]. The article that presented the highest AUC (0.88) evaluated the probability of TKA within 5 years [47].

### Support vector machine

SVM is an effective model which can be used for both classification and regression; developed in the 1960s it still is one of the most popular algorithms used to classify disease progression based on imaging data. However, due to its low accuracy in performances with noisy datasets, newly developed algorithms such as K-Nearest Neighbors (kNN) are gaining prominence in this role. SVM is particularly effective when the number of features exceeds the number of samples in the data, being able to handle both linear and non-linear relationships in data. It was used by 13 articles, one of them evaluating the prediction of LOS and complications after TKA [29, 51]. Mainly to assess clinical outcomes such as: prolonged postoperative opioid prescription [32]; improvement of KOOS one year after TKA [33]; dissatisfaction after TKA [6]; attainment of MCIDs 2 years after TKA [20, 53]. SVM was also employed to analyze subtask segmentation of the TUG test for perioperative TKA [24]; Risk and Time of TKA in patients with knee OA [16, 27]; surgical corrections based on patient-specific intra-operative evaluation [49]. Additionally, one article used the algorithm to evaluate the characterization of tissues [15, 60] while another applied SVM in component sizing for TKA [35].

### Other AI models

Two AI models were employed to evaluate major complications after primary TKA [17]: AutoPrognosis (AP) and AdaBoost. The ML method Decision tree was utilized in two studies for the analysis of the following outputs: gait comparison between UKA and TKA patient [30], and subtask segmentation of TUG test for perioperative TKA, the latter also being assessed by the methods: AdaBoost, kNN, Naïve Bayes Classifier (NB) [24].

Regarding the analysis of post-TKA walking limitation, the model SuperLearner was used [7]. Both the Cox-PH model and DeepSurv model were used to predict the risk and time of TKA in patients with knee osteoarthritis [27]; an Ensemble Deep Learning (DL) model based on the use of MRI and radiograph was also compared with traditional ML algorithms to predict the risk of TKA, obtaining promising results [40]. The prediction of PROs was assessed by the models: NB, kNN, and Multi-Step Adaptive Elastic-Net (MSAENET) [26].

The models Quadratic Discriminant Analysis (QDA) and LASSO regression were employed to evaluate MCIDs attainment after TKA in different periods. One of the studies made the assessment 1 year after TKA [22], other two articles made the evaluation 2 years after TKA [20, 53]. LASSO regression was also used to analyze mortality and complication after TKA, such as respiratory, cardiovascular, and nervous system and renal complications [21]. Regarding the prediction of clinical outcomes, the new Skeletal Oncology Research Group Machine Learning Algorithm (SORG-MLA) was validated for the identification of patients at risk of prolonged postoperative opioid use after TKA, obtaining an AUC: 0.75 [48].

Moreover, the models Linear Discriminant Analysis (LDA), Recursive Partitioning (RP), and NB were employed for the assessment of sensitization in patients with chronic pain after TKA [1]. The prediction of procedural cost after TKA, the DenseNet was used, presenting an AUC score of 0.813 [31].

Natural Language Processing Method (NLPM) was utilized to assess surgical technique, using the following outputs: category of surgery, implant model, presence of patellar resurfacing, constraint type, and laterality of surgery [46]. NLPM was also used to estimate ITS data [4] and analyze the alteration that opioid use can have in risk of knee revision and manipulation in the first year after primary TKA [12].

Lastly, the Stochastic Hill Climbing Complexity score was for the prediction of surgical 90-day morbidity, mortality, and complications [11]. NB was employed to analyze inpatient cost and LOS after TKA [32, 45].

### Quality assessment by modified MINORS

All 49 of the reviewed articles were evaluated following the modified MINORS checklist to assess quality and risk of bias. All 49 articles clearly reported the study aim, however, 11 studies failed to report the performance metric. Two publications did not report the output feature, while 46 of the studies clearly stated the input feature, and 45 of the articles indicated disclosure. Regarding the item AI model, 45 of the reviewed articles fulfilled this criterion. These findings showed a relatively high degree of quality and low likelihood of bias, only two of the reviewed articles received a score of 5/8, five articles with 6/8 as a score, and the majority, 42 out of 49 publications, scored 7/8 and higher (Table 5).

**Table 5:** Quality assessment by modified MINORS

**Author, year**	**Disclosure**	**Study Aim**	**Input feature**	**Output feature**	**Validation method**	**Dataset distribution**	**Performance metric**	**AI model**	*Score*
Ben-Ari et al., 2017 [12]	1	1	1	1	1	0	1	0	6
Bloomfield et al., 2019 [13]	1	1	1	1	0	1	0	1	6
Bonakdari et al., 2020 [10]	1	1	0	0	1	1	0	1	5
Bovonratwet et al., 2021 [14]	1	1	1	1	0	1	1	0	6
Chan et al., 2020 [15]	1	1	0	1	1	1	1	1	7
Crawford et al., 2023 [16]	1	1	1	1	1	0	1	1	7
Devana et al., 2021 [17]	1	1	1	1	1	1	1	1	8
Farooq et al., 2020 [18]	1	1	1	0	1	1	1	1	7
Farooq et al., 2021 [19]	1	1	1	1	1	1	0	1	7
Fontana et al., 2019 [20]	1	1	1	1	1	1	0	1	7
Harris et al., 2019 [21]	1	1	1	1	1	1	1	1	8
Harris et al., 2021 [22]	1	1	1	1	1	1	1	1	8
Heisinger et al., 2020 [23]	1	1	1	1	1	1	0	1	7
Hinterwimmer et al., 2022 [5]	1	1	1	1	1	1	1	1	8
Hsieh et al., 2020 [24]	1	1	1	1	1	1	1	1	8
Huang et al., 2018 [25]	1	1	1	1	1	0	1	1	7
Huber et al., 2019 [26]	1	1	1	1	1	1	1	1	8
Hyer et al., 2020 [11]	1	1	1	1	1	1	1	1	8
Jamshidi et al., 2021 [27]	1	1	1	1	1	1	1	1	8
Jayakumar et al., 2021 [28]	1	1	1	1	1	1	0	0	6
Jo et al., 2020 [8]	1	1	1	1	1	0	1	1	7
Johannesdottir et al., 2022 [29]	1	1	1	1	1	1	1	1	8
Jones et al., 2016 [30]	1	1	1	1	1	1	0	1	7
Karnuta et al., 2019 [31]	0	1	1	1	1	1	1	1	7
Katakam et al., 2020 [32]	1	1	1	1	1	1	1	1	8
Katakam et al., 2022 [33]	1	1	1	1	1	1	1	1	8
Kunze et al., 2020 [6]	0	1	1	1	1	1	1	1	7
Kunze et al., 2021 [35]	1	1	1	1	0	1	1	1	7
Leung et al., 2020 [36]	1	1	1	1	1	0	1	1	7
Li et al., 2022 [37]	1	1	1	1	1	1	1	1	8
Mohammed et al., 2022 [38]	0	1	1	1	1	1	1	1	7
Navarro et al., 2018 [39]	1	1	1	1	1	1	1	1	8
Pua et al., 2020 [7]	1	1	1	1	1	1	1	1	8
Rajamohan et al., 2023 [40]	1	1	1	1	1	1	1	1	8
Ramazanian et al., 2022 [41]	1	1	1	1	0	1	0	0	5
Ramkumar et al., 2019 [42]	1	1	1	1	1	1	1	1	8
Ramkumar et al., 2019 [43]	1	1	1	1	1	1	0	1	7
Rexwinkle et al., 2018 [44]	0	1	1	1	1	1	0	1	6
Sachau et al., 2022 [45]	1	1	1	1	1	1	0	1	7
Sagheb et al., 2021 [46]	1	1	0	1	1	1	1	1	7
Shohat et al., 2020 [4]	1	1	1	1	1	1	1	1	8
Tolpadi et al., 2020 [47]	1	1	1	1	1	1	1	1	8
Tsai et al., 2023 [48]	1	1	1	1	1	1	1	1	8
Verstraete et al., 2020 [49]	1	1	1	1	1	1	1	1	8
Wei et al., 2021 [50]	1	1	1	1	1	1	1	1	8
Yeo et al., 2023 [51]	1	1	1	1	0	1	1	1	7
Yi et al., 2020 [52]	1	1	1	1	1	1	1	1	8
Zhang et al., 2022 [53]	1	1	1	1	1	1	1	1	8
*Count*	45	49	46	47	44	44	38	45	

## Discussion

This systematic review evaluated the possible uses of AI/ML models in TKA, highlighting their potential in improving decision-making, component sizing, inpatient costs, perioperative planning, and streamlining the surgical workflow. Implementing these prediction models in TKA can ultimately lead to more accurate predictions, less time-consuming data processing, and higher precision in identifying patterns, all while minimizing user input bias to provide risk-based patient-specific care.

A key finding was the benefits of RF in aiding surgical decision-making when applied in intraoperatively collected surface models and patient-specific intraoperative assessments. RF outperformed both ANN and SVM not only when categorizing various types of anatomical tissue [15], but also when identifying populations at risk for TKA [16], and assessing balance and alignment during TKA surgery, aiding the surgeon regarding the optimal choice for the suitable bone recut or soft tissue adjustment [49, 61]. This review highlights how the application of RF in all the steps leading to TKA, perioperative and postoperative care can lead to optimal clinical and surgical outcomes, while reducing complications thanks to patient-specific planning. Moreover, by streamlining the surgical workflow and helping to select surgical corrections, this AI model can overcome the risk of data overload and the challenge of data interpretation, while being fast, cost-efficient, and accurate.

The SGB model presented promising results in the Kunze et al. (2021) study, by outranking RF, SVM, and EPLR for the prediction of the component sizing of the implant used in TKA. This model demonstrated the best overall performance regarding minimizing prediction error and maximizing accuracy for both femoral and tibial implant component size prediction. A potential benefit is an ability to predict final component sizes of the prosthetic without reliance on digital or manual templating, therefore being faster than traditional methods. Also, showing good performance across different TKA component manufacturers, streamlining component selection processes, improving inventory control, and reducing shipping costs [35, 62].

Regarding prediction models for allogenic blood transfusion, the highest AUC score was reported by the RF and SVM-based models [25]. With a slightly lower difference of 0.038 in the AUC score, the ANN-based model was still significantly higher than the classic prediction models [38]. Overall, these results show how the implementation of various ML-based models can result in an improvement of peri-operative complications predictions, ensuring that the identified population at risk, for blood transfusion, receives proper care while also optimizing the operative process and reducing the risk of prolonged LOS, caused by complications, such as blood transfusion, during TKA.

A further finding is the already established importance of LR models when used in healthcare settings, which can lead to the development of patient-specific care and peri-operative planning. The most successful result of LR (AUC 0.88) was achieved by its implementation, together with DenseNet, in identifying a population at higher risk of TKA within 5 years, particularly at less advanced stages of OA [47]; although, in the more recent study published by Crawford et al. in 2023, compared to other models such as SGB and RF (AUC: 0.83), EPLR scored a lower performance in identification of population at risk of TKA [16]. Additionally, implementing LR with other models, like the ML-based remote patient monitoring system, can reduce the need for TKA revision, while acquiring continuous data for patients undergoing TKA, in terms of mobility and rehabilitation compliance. This patient monitoring system proved to be reliable, low-maintenance, and a well-received platform for the patient recovering from TKA [42]. Implementing LR models would result in higher objectivity, cost-effectiveness, and ability to acquire continuous data, together with higher accuracy in identifying at-risk population, overall increasing the success rate for TKA.

Financial aspects are to be considered when proposing a treatment plan to patients, as complications can arise during the surgery and recovery, drastically changing the cost expected beforehand. Although it was shown to be an important element to consider when planning peri-operative care during TKA, the cost-prediction outcome was only analysed in one article. Demonstrating high accuracy when used in clinical medicine, the DenseNet model [31, 63] can optimize and provide a cost-efficient organization of resources that can benefit the medical staff by reducing their workload and improving the quality of the arrangement of resources. Simultaneously, this method can identify populations at risk for complications, a benefit that would help reduce the higher cost of the procedure after TKA, making it possible to implement patient-specific payment plans benefitting both patients and healthcare providers.

Going over the performances of the GBM model analysed in different articles, we can observe how this algortihm is simple and efficient, it has been validated to improve both short- and long-term prognoses of TKA patients. Ko et al. successfully used this AI model for the prediction of the development of postoperative AKI after TKA, which can not only increase LOS but also be life-threatening [34]; while TreeNet GBM proved to be the most successful method when applied for predictors regarding patient satisfaction [18]. Additionally, GBM showed great results when predicting the disposition of patients at discharge [38], therefore the model’s implementation could improve the overall patient satisfaction and recovery rate post-TKA, while also assuring patient-specific peri-operative care is applied to prevent and manage possible complications.

Looking at more novel models less implemented up until recently in the healthcare settings, the following AI/ML models: DL-TL-MT, SVM, Deep Surv, and Cox-PH, proved to be of great use to individuate the population of patients at risk and develop patient-specific care. The DL-TL-MT model successfully predicted the risk of OA progression based on knee radiographs in patients that previously underwent TKA [36]. Presenting the same AUC level (of 0.87), the methods SVM, Deep Surv, and Cox-PH were successfully employed to predict the risk and time of TKA of an OA knee [27]. The implementation methods prove to be indispensable in predicting the progression of OA, even at an early stage. This ML-based model has great potential as a diagnostic tool for physicians when determining the prognosis for patient at all stages of OA, allowing for early intervention through TKA where needed, therefore reducing the risk of complications and of TKA revision.

The SVM predictor model showed also a very promising results when applied in the different settings, and especially for the segmentation of the TUG test and extraction of information from each subtask perioperative to TKA, solving the problems regarding subjectiveness and other biases [24, 64]. The benefits that come with the usage of this AI model would be a more precise segmentation and therefore data extraction, which results in further understanding and classification of improvements in patients, leading to the employment of patient-specific treatments and rehabilitation models.

Looking at the results of the different articles involved in the review, the emergence of ML models in the medical setting becomes an evident matter: most data corroborates the idea that novel AI models present better results and predictive powers, compared to traditional models, when identifying predictors of TKA and analyzing multiple outcomes simultaneously. In the prediction of complications after primary TKA, Devana et al. prove the superiority of AP, compared to traditional models, regarding the discriminative ability and the capability to suggest nonlinear relationships between variables in the outcomes of TKA. Consequently, AP can be a versatile tool that may be utilized for the identification of crucial patient characteristics when predicting outcomes across a variety of datasets, thereby improving the patient outcomes [17]. Additionally, Harris et al. demonstrated how AI can produce preoperative prediction models for one-year improvement in pain and functioning after TKA; and how the GBM model, which performs well in important interactions, and the QDA model, which performs better in nonlinear association, can be applied to produce an easy-to-use predictive model able to achieve similar or better accuracy with far fewer inputs in respect to traditional predictive models [22].

Lastly, the NLPM model presents great potential as a newly emerging algorithm, in particular when applied in clinical settings for the interpretation of a text, which has been applied in different studies for the classification of patient satisfaction [14], knee revisions after TKA due to preoperative opioid use [12], and for the processing of clinical free text from electronic health records [46]. The strength of this ML-based model relies on its ability to automate the extraction of embedded information in perioperative notes and patient-centered surveys, decreasing the need for costly manual chart reviewing and improving data quality while being less time-consuming. The use of this model would improve patient feedback and perioperative notes to better patient-specific risk-based care resulting in higher patient satisfaction and a reduction in costs for the healthcare system due to possible lawsuits [65], together with the reduction of the cost due to manual chart reviews [46].

Like both the Hinterwimmer et al. 2021, and the Lee et al. 2022 review, this systematic review confirms the great potential of AI/ML methods and their application in orthopedics for cost predictions, diagnostic applications, and identification of risk factors, while also clearing the doubts regarding the inaccuracy and lack of sufficient evaluation of these models. In comparison, this review analyzed 49 articles, including the publications already examined in previous reviews. This more extensive research concluded that not only is it possible to implement these models in the prediction of TKA perioperative care, disease progression of OA, and distinct outcomes applying specific data, but also the prediction of more complex outcomes is now feasible through the application of more novel AI/ML algorithms [13, 17, 21, 22, 27, 30]. Although, as mentioned in several studies, further research may enhance the reliability of AI/ML models and allow for their use in patient preoperative and perioperative care [8, 11, 19, 21, 43, 50].

### Limitations

The main limitation of this review derives from the possible bias of information regarding the performance of the different AI models, which, as highlighted by the MINORS table, results as the most at-risk parameter due to the omission by several articles of either AUC score or Accuracy score for the different predictive models examined. Moreover, many of the studies included in this review are retrospective studies obtaining the data, regarding the patients for the testing of the AI/ML prediction models, from national databases and electronic health recordings; limitations by the lack of detailed clinical information, potential misclassification of data, and in many cases a small cohort of patients presenting limited characteristics from which to derive input and compare outputs, which may lead to the results not being generalizable to all patient populations [11, 19, 21]. Validation of analyzed predictive models on larger populations of patients is needed. Lastly, due to the heterogeneity between data, it was not possible to perform a meta-analysis.

## Conclusion

Regarding the implementation of AI/ML models in TKA, the articles in this review mostly consider these predictive models to be helpful and suggest that their application in medical settings for perioperative TKA clinical decision-making and prediction of the progression of OA into TKA may result in an improvement of patient satisfaction, risk managing, and cost efficiency. Among the best qualities, for which the AI/ML models outperform the traditional prediction models, frequently reported higher accuracy, cost efficiency, simple application, lack of subjectiveness, and overall reduction of time consumption thanks to the automation of tasks. Therefore, it is possible to conclude that, although the results of the reviewed articles should be further validated by their testing on larger cohorts of patients, the findings of these articles highlight the great potentials that derive from the inclusion of AI/ML predictive models in a further branch of medicine.

## Data Availability

The datasets used and/or analyzed during the current study are available from the corresponding author on reasonable request.

## Abbreviations

AdaBoost: Adaptive Boosting
AI: Artificial Intelligence
AKI: Acute Kidney Infection
ALBT: Allogenic Blood Transfusion
ANN: Artificial Neural Network
ANN-TL-MT: Artificial Neural Network – Transfer Learning – multitask
AP: AutoPrognosis
APR: All patient refined
ASA-PS: American society of anesthesiologists’ physical status
ASA: America Society of Anesthesiologists
AUC-ROC: Area Under the Receiver Operating Characteristic Curve
BMI: Body mass index
BP: Blood pressure
BT: Blood transfusion
CBC: Cartilage and bone classification
CCS: Charlson comorbidity score
CMS: Centers for Medicare & Medicaids Services
CoxPH: Cox proportional hazards
CPS: Cohort pilot study
CS: Comparative study
DCNN: Deep Convolutional Neural Network
DD: Discharge disposition
DenseNet: Densely Connected Convolutional Network
DL: Deep Learning
DP: Disposition of patient
DS: DeepSurv
DT: Decision tree
EPLR: Elastic-net Penalized Logistic Regression
ESSKA: European Society of Sports Traumatology, Knee Surgery and Arthroscopy
GBM: Gradient Boosting Machine
Hb: Heamoglobin
HEP: Home exercise program
HKA: Hip-knee-angle
IC: Impatient charges/costs
KA: Knee Artrhoplasty
KNN: K-Nearest Neighbors
KOOS: Knee Injury and Osteoarthritis Outcome Score
KOOS-JR: Knee injury and Osteoarthritis Outcome Score for Joint Replacement
KSS-F: Knee society score function
KSS: Knee society score
LASSO: Least Absolute Shrinkage and Selection Operator
LDA: Linear Discriminant Analysis
LOS: Length of stay
LR: Logistic Regression
MCBC: Muscle, cartilage, bone classification
MCIDs: Minimally clinically important differences
MCRS: Multi center retrospective study
MCS: Mechanical circulatory support
MINORS: Methodological Index for Non-Randomized Studies
ML: Machine Learning
MLP: Multilayer perceptron
MSAENET: Multi-Step Adaptive Elastic NETwork
MTBCC: Muscle, tendon, bone, cartilage classification
MTLR: Multi-Task Logistic Regression
NB: Näive-Bayes
NHS: National Health Service
NIS: National (Nationwide) Inpatient Sample database
NLPM: Natural Language Processing Method
NN: Neural Network
Non-Linear-GMDH: Non-Linear-Group Method of Data Handling
NSAID: Non-steroidal anti-inflammatory drugs
NSQIP: National Surgical Quality Improvement Program
OA: Osteoarthritis
OAI: Osteoarthritis initiative
OME: Orthopedic Minimal Data Set database
OSHPD: Office of Statewide Health Planning and Development
PASE: Physical activity scale for the Elderly
PCL: Posterior cruciate ligament
PCS: Physical component summary
PHRS: Preoperative patient-reported health state
PICO: Population Intervention Comparison Outcome
PJI: Prosthetic Joint Infection
PRISMA: Preferred Reporting Items for Systematic reviews and Meta-Analysis
PROMs: Patient reported outcome measures
PROs: Prediction of patient-reported outcomes
PS: Pilot study
QDA: Quadrant Discriminant Analysis
QOL: Quality of Life
RCS: Retrospective cohort study
RCT: Randomized control trial
RF: Random Forest
RP: Recursive Partitioning
RPM: Remote Patient Monitoring
SAFs: Standard analytical files
SF-36 PCS: Short form – physical component summary
SGB: Stochastic Gradient Boosting
SHC: Stochastic Hill Climbing
SORG-MLA: Skeletal Oncology Research Group Machine Learning Algorithm
SPARCS: State-wide Planning and Research Cooperative System
SVM: Support Vector Machines
TKA: Total Knee Arthroplasty
TUG: Timed Up-and-Go
UCLA: University of California Los Angeles
UKA: Unicompartmental Knee Arthroplasty
VA: Veteran’s affairs
VAS: Visual analog scale
WHO: World Health Organization
WOMAC: Western Ontario and McMaster Universities Osteoarthritis Index
XGBoost: EXtreme Gradient Boosting

## References

[1] Cabitza F, Locoro A, Banfi G. Machine learning in orthopedics: a literature review. Front Bioeng Biotechnol. 2018;6:75. 29998104 10.3389/fbioe.2018.00075 PMC6030383

[2] Lee LS, Chan PK, Wen C, Fung WC, Cheung A, Chan VWK, Cheung MH, Fu H, Yan CH, Chiu KY. Artificial intelligence in diagnosis of knee osteoarthritis and prediction of arthroplasty outcomes: a review. Arthroplasty. 2022;4(1):16. 35246270 10.1186/s42836-022-00118-7 PMC8897859

[3] Martín Noguerol T, Paulano-Godino F, Martín-Valdivia MT, Menias CO, Luna A. Strengths, weaknesses, opportunities, and threats analysis of artificial intelligence and machine learning applications in radiology. J Am Coll Radiol. 2019;16(9 Pt B):1239–47. 31492401 10.1016/j.jacr.2019.05.047

[4] Shohat N, Goswami K, Tan TL, Yayac M, Soriano A, Sousa R, Wouthuyzen-Bakker M, Parvizi J. (NINJA) ESGoIAIEatNINoJA: 2020 Frank Stinchfield award: identifying who will fail following irrigation and debridement for prosthetic joint infection. Bone Joint J. 2020;102-B(7_Supple_B):11–9. 32600194 10.1302/0301-620X.102B7.BJJ-2019-1628.R1

[5] Hinterwimmer F, Lazic I, Suren C, Hirschmann MT, Pohlig F, Rueckert D, Burgkart R, von Eisenhart-Rothe R. Machine learning in knee arthroplasty: specific data are key-a systematic review. Knee Surg Sports Traumatol Arthrosc. 2022;30(2):376–88. 35006281 10.1007/s00167-021-06848-6 PMC8866371

[6] Kunze KN, Polce EM, Sadauskas AJ, Levine BR. Development of machine learning algorithms to predict patient dissatisfaction after primary total knee arthroplasty. J Arthroplasty. 2020;35(11):3117–22. 32564970 10.1016/j.arth.2020.05.061

[7] Pua YH, Kang H, Thumboo J, Clark RA, Chew ES, Poon CL, Chong HC, Yeo SJ. Machine learning methods are comparable to logistic regression techniques in predicting severe walking limitation following total knee arthroplasty. Knee Surg Sports Traumatol Arthrosc. 2020;28(10):3207–16. 31832697 10.1007/s00167-019-05822-7

[8] Jo C, Ko S, Shin WC, Han HS, Lee MC, Ko T, Ro DH. Transfusion after total knee arthroplasty can be predicted using the machine learning algorithm. Knee Surg Sports Traumatol Arthrosc. 2020;28(6):1757–64. 31254027 10.1007/s00167-019-05602-3

[9] Ogink PT, Groot OQ, Karhade AV, Bongers MER, Oner FC, Verlaan JJ, Schwab JH. Wide range of applications for machine-learning prediction models in orthopedic surgical outcome: a systematic review. Acta Orthop. 2021;92(5):526–31. 34109892 10.1080/17453674.2021.1932928 PMC8519550

[10] Bonakdari H, Pelletier JP, Martel-Pelletier J. A reliable time-series method for predicting arthritic disease outcomes: new step from regression toward a nonlinear artificial intelligence method. Comput Methods Programs Biomed. 2020;189:105315. 31972347 10.1016/j.cmpb.2020.105315

[11] Hyer JM, White S, Cloyd J, Dillhoff M, Tsung A, Pawlik TM, Ejaz A. Can we improve prediction of adverse surgical outcomes? Development of a surgical complexity score using a novel machine learning technique. J Am Coll Surg. 2020;230(1):43–52.e41. 31672674 10.1016/j.jamcollsurg.2019.09.015

[12] Ben-Ari A, Chansky H, Rozet I. Preoperative opioid use is associated with early revision after total knee arthroplasty: a study of male patients treated in the veterans affairs system. J Bone Joint Surg Am. 2017;99(1):1–9. 28060227 10.2106/JBJS.16.00167

[13] Bloomfield RA, Williams HA, Broberg JS, Lanting BA, McIsaac KA, Teeter MG. Machine learning groups patients by early functional improvement likelihood based on wearable sensor instrumented preoperative timed-up-and-go tests. J Arthroplasty. 2019;34(10):2267–71. 31255408 10.1016/j.arth.2019.05.061

[14] Bovonratwet P, Shen TS, Islam W, Ast MP, Haas SB, Su EP. Natural language processing of patient-experience comments after primary total knee arthroplasty. J Arthroplasty. 2021;36(3):927–34. 33127238 10.1016/j.arth.2020.09.055

[15] Chan B, Rudan JF, Mousavi P, Kunz M. Intraoperative integration of structured light scanning for automatic tissue classification: a feasibility study. Int J Comput Assist Radiol Surg. 2020;15(4):641–9. 32144629 10.1007/s11548-020-02129-8

[16] Crawford AM, Karhade AV, Agaronnik ND, Lightsey HM, Xiong GX, Schwab JH, Schoenfeld AJ, Simpson AK. Development of a machine learning algorithm to identify surgical candidates for hip and knee arthroplasty without in-person evaluation. Arch Orthop Trauma Surg. 2023;143(9):5985–92. 36905425 10.1007/s00402-023-04827-9 PMC10008010

[17] Devana SK, Shah AA, Lee C, Roney AR, van der Schaar M, SooHoo NF. A novel, potentially universal machine learning algorithm to predict complications in total knee arthroplasty. Arthroplast Today. 2021;10:135–43. 34401416 10.1016/j.artd.2021.06.020 PMC8349766

[18] Farooq H, Deckard ER, Ziemba-Davis M, Madsen A, Meneghini RM. Predictors of patient satisfaction following primary total knee arthroplasty: results from a traditional statistical model and a machine learning algorithm. J Arthroplasty. 2020;35(11):3123–30. 32595003 10.1016/j.arth.2020.05.077

[19] Farooq H, Deckard ER, Arnold NR, Meneghini RM. Machine learning algorithms identify optimal sagittal component position in total knee arthroplasty. J Arthroplasty. 2021;36(7S):S242–9. 33744081 10.1016/j.arth.2021.02.063

[20] Fontana MA, Lyman S, Sarker GK, Padgett DE, MacLean CH. Can machine learning algorithms predict which patients will achieve minimally clinically important differences from total joint arthroplasty? Clin Orthop Relat Res. 2019;477(6):1267–79. 31094833 10.1097/CORR.0000000000000687 PMC6554103

[21] Harris AHS, Kuo AC, Weng Y, Trickey AW, Bowe T, Giori NJ. Can machine learning methods produce accurate and easy-to-use prediction models of 30-day complications and mortality after knee or hip arthroplasty? Clin Orthop Relat Res. 2019;477(2):452–60. 30624314 10.1097/CORR.0000000000000601 PMC6370104

[22] Harris AHS, Kuo AC, Bowe TR, Manfredi L, Lalani NF, Giori NJ. Can machine learning methods produce accurate and easy-to-use preoperative prediction models of one-year improvements in pain and functioning after knee arthroplasty? J Arthroplasty. 2021;36(1):112–117.e116. 32798181 10.1016/j.arth.2020.07.026

[23] Heisinger S, Hitzl W, Hobusch GM, Windhager R, Cotofana S. Predicting total knee replacement from symptomology and radiographic structural change using artificial neural networks-data from the osteoarthritis initiative (OAI). J Clin Med. 2020;9(5):1298. 32369985 10.3390/jcm9051298 PMC7288322

[24] Hsieh CY, Huang HY, Liu KC, Chen KH, Hsu SJ, Chan CT. Subtask segmentation of timed up and go test for mobility assessment of perioperative total knee arthroplasty. Sensors (Basel). 2020;20(21):6302. 33167444 10.3390/s20216302 PMC7663910

[25] Huang Z, Huang C, Xie J, Ma J, Cao G, Huang Q, Shen B, Byers Kraus V, Pei F. Analysis of a large data set to identify predictors of blood transfusion in primary total hip and knee arthroplasty. Transfusion. 2018;58(8):1855–62. 30145838 10.1111/trf.14783 PMC6131039

[26] Huber M, Kurz C, Leidl R. Predicting patient-reported outcomes following hip and knee replacement surgery using supervised machine learning. BMC Med Inform Decis Mak. 2019;19(1):3. 30621670 10.1186/s12911-018-0731-6 PMC6325823

[27] Jamshidi A, Pelletier JP, Labbe A, Abram F, Martel-Pelletier J, Droit A. Machine learning-based individualized survival prediction model for total knee replacement in osteoarthritis: data from the osteoarthritis initiative. Arthritis Care Res (Hoboken). 2021;73(10):1518–27. 33749148 10.1002/acr.24601

[28] Jayakumar P, Moore MG, Furlough KA, Uhler LM, Andrawis JP, Koenig KM, Aksan N, Rathouz PJ, Bozic KJ. Comparison of an artificial intelligence-enabled patient decision aid vs educational material on decision quality, shared decision-making, patient experience, and functional outcomes in adults with knee osteoarthritis: a randomized clinical trial. JAMA Netw Open. 2021;4(2):e2037107. 33599773 10.1001/jamanetworkopen.2020.37107 PMC7893500

[29] Johannesdottir KB, Kehlet H, Petersen PB, Aasvang EK, Sørensen HBD, Jørgensen CC, Group CfF-tHaKRC. Machine learning classifiers do not improve prediction of hospitalization > 2 days after fast-track hip and knee arthroplasty compared with a classical statistical risk model. Acta Orthop. 2022;93:117–23. 34984485 10.2340/17453674.2021.843 PMC8815306

[30] Jones GG, Kotti M, Wiik AV, Collins R, Brevadt MJ, Strachan RK, Cobb JP. Gait comparison of unicompartmental and total knee arthroplasties with healthy controls. Bone Joint J. 2016;98-B(10 Supple B):16–21. 27694511 10.1302/0301-620X.98B10.BJJ.2016.0473.R1 PMC5047137

[31] Karnuta JM, Navarro SM, Haeberle HS, Helm JM, Kamath AF, Schaffer JL, Krebs VE, Ramkumar PN. Predicting inpatient payments prior to lower extremity arthroplasty using deep learning: which model architecture is best? J Arthroplasty. 2019;34(10):2235-2241.e2231. 31230954 10.1016/j.arth.2019.05.048

[32] Katakam A, Karhade AV, Schwab JH, Chen AF, Bedair HS. Development and validation of machine learning algorithms for postoperative opioid prescriptions after TKA. J Orthop. 2020;22:95–9. 32300270 10.1016/j.jor.2020.03.052 PMC7152687

[33] Katakam A, Karhade AV, Collins A, Shin D, Bragdon C, Chen AF, Melnic CM, Schwab JH, Bedair HS. Development of machine learning algorithms to predict achievement of minimal clinically important difference for the KOOS-PS following total knee arthroplasty. J Orthop Res. 2022;40(4):808–15. 34275163 10.1002/jor.25125

[34] Ko S, Jo C, Chang CB, Lee YS, Moon YW, Youm JW, Han HS, Lee MC, Lee H, Ro DH. A web-based machine-learning algorithm predicting postoperative acute kidney injury after total knee arthroplasty. Knee Surg Sports Traumatol Arthrosc. 2022;30(2):545–54. 32880677 10.1007/s00167-020-06258-0

[35] Kunze KN, Polce EM, Patel A, Courtney PM, Levine BR. Validation and performance of a machine-learning derived prediction guide for total knee arthroplasty component sizing. Arch Orthop Trauma Surg. 2021;141(12):2235–44. 34255175 10.1007/s00402-021-04041-5

[36] Leung K, Zhang B, Tan J, Shen Y, Geras KJ, Babb JS, Cho K, Chang G, Deniz CM. Prediction of total knee replacement and diagnosis of osteoarthritis by using deep learning on knee radiographs: data from the osteoarthritis initiative. Radiology. 2020;296(3):584–93. 32573386 10.1148/radiol.2020192091 PMC7434649

[37] Li H, Jiao J, Zhang S, Tang H, Qu X, Yue B. Construction and comparison of predictive models for length of stay after total knee arthroplasty: regression model and machine learning analysis based on 1,826 cases in a single Singapore center. J Knee Surg. 2022;35(1):7–14. 32512596 10.1055/s-0040-1710573

[38] Mohammed H, Huang Y, Memtsoudis S, Parks M, Ma Y. Utilization of machine learning methods for predicting surgical outcomes after total knee arthroplasty. PLoS One. 2022;17(3):e0263897. 35316270 10.1371/journal.pone.0263897 PMC8939835

[39] Navarro SM, Wang EY, Haeberle HS, Mont MA, Krebs VE, Patterson BM, Ramkumar PN. Machine learning and primary total knee arthroplasty: patient forecasting for a patient-specific payment model. J Arthroplasty. 2018;33(12):3617–23. 30243882 10.1016/j.arth.2018.08.028

[40] Rajamohan HR, Wang T, Leung K, Chang G, Cho K, Kijowski R, Deniz CM. Prediction of total knee replacement using deep learning analysis of knee MRI. Sci Rep. 2023;13(1):6922. 37117260 10.1038/s41598-023-33934-1 PMC10147603

[41] Ramazanian T, Yan S, Rouzrokh P, Wyles CC, O Byrne TJ, Taunton MJ, Maradit Kremers H. Distribution and correlates of hip-knee-ankle angle in early osteoarthritis and preoperative total knee arthroplasty patients. J Arthroplasty. 2022;37(6S):S170–5. 35210147 10.1016/j.arth.2021.12.009 PMC9117418

[42] Ramkumar PN, Haeberle HS, Ramanathan D, Cantrell WA, Navarro SM, Mont MA, Bloomfield M, Patterson BM. Remote patient monitoring using mobile health for total knee arthroplasty: validation of a wearable and machine learning-based surveillance platform. J Arthroplasty. 2019;34(10):2253–9. 31128890 10.1016/j.arth.2019.05.021

[43] Ramkumar PN, Karnuta JM, Navarro SM, Haeberle HS, Scuderi GR, Mont MA, Krebs VE, Patterson BM. Deep learning preoperatively predicts value metrics for primary total knee arthroplasty: development and validation of an artificial neural network model. J Arthroplasty. 2019;34(10):2220–2227.e2221. 31285089 10.1016/j.arth.2019.05.034

[44] Rexwinkle JT, Werner NC, Stoker AM, Salim M, Pfeiffer FM. Investigating the relationship between proteomic, compositional, and histologic biomarkers and cartilage biomechanics using artificial neural networks. J Biomech. 2018;80:136–43. 30269929 10.1016/j.jbiomech.2018.08.032

[45] Sachau J, Otto JC, Kirchhofer V, Larsen JB, Kennes LN, Hüllemann P, Arendt-Nielsen L, Baron R. Development of a bedside tool-kit for assessing sensitization in patients with chronic osteoarthritis knee pain or chronic knee pain after total knee replacement. Pain. 2022;163(2):308–18. 33990109 10.1097/j.pain.0000000000002335

[46] Sagheb E, Ramazanian T, Tafti AP, Fu S, Kremers WK, Berry DJ, Lewallen DG, Sohn S, Maradit Kremers H. Use of natural language processing algorithms to identify common data elements in operative notes for knee arthroplasty. J Arthroplasty. 2021;36(3):922–6. 33051119 10.1016/j.arth.2020.09.029 PMC7897213

[47] Tolpadi AA, Lee JJ, Pedoia V, Majumdar S. Deep learning predicts total knee replacement from magnetic resonance images. Sci Rep. 2020;10(1):6371. 32286452 10.1038/s41598-020-63395-9 PMC7156761

[48] Tsai CC, Huang CC, Lin CW, Ogink PT, Su CC, Chen SF, Yen MH, Verlaan JJ, Schwab JH, Wang CT, et al. The Skeletal Oncology Research Group Machine Learning Algorithm (SORG-MLA) for predicting prolonged postoperative opioid prescription after total knee arthroplasty: an international validation study using 3,495 patients from a Taiwanese cohort. BMC Musculoskelet Disord. 2023;24(1):553. 37408033 10.1186/s12891-023-06667-5 PMC10320986

[49] Verstraete MA, Moore RE, Roche M, Conditt MA. The application of machine learning to balance a total knee arthroplasty. Bone Jt Open. 2020;1(6):236–44. 33225295 10.1302/2633-1462.16.BJO-2020-0056.R1 PMC7677727

[50] Wei C, Quan T, Wang KY, Gu A, Fassihi SC, Kahlenberg CA, Malahias MA, Liu J, Thakkar S, Gonzalez Della Valle A, et al. Artificial neural network prediction of same-day discharge following primary total knee arthroplasty based on preoperative and intraoperative variables. Bone Joint J. 2021;103(8):1358–66. 34334050 10.1302/0301-620X.103B8.BJJ-2020-1013.R2

[51] Yeo I, Klemt C, Robinson MG, Esposito JG, Uzosike AC, Kwon YM. The use of artificial neural networks for the prediction of surgical site infection following TKA. J Knee Surg. 2023;36(6):637–43. 35016246 10.1055/s-0041-1741396

[52] Yi PH, Wei J, Kim TK, Sair HI, Hui FK, Hager GD, Fritz J, Oni JK. Automated detection & classification of knee arthroplasty using deep learning. Knee. 2020;27(2):535–42. 31883760 10.1016/j.knee.2019.11.020

[53] Zhang S, Lau BPH, Ng YH, Wang X, Chua W. Machine learning algorithms do not outperform preoperative thresholds in predicting clinically meaningful improvements after total knee arthroplasty. Knee Surg Sports Traumatol Arthrosc. 2022;30(8):2624–30. 34245310 10.1007/s00167-021-06642-4

[54] Longo UG, De Salvatore S, Intermesoli G, Pirato F, Piergentili I, Becker R, Denaro V. Metaphyseal cones and sleeves are similar in improving short- and mid-term outcomes in total knee arthroplasty revisions. Knee Surg Sports Traumatol Arthrosc. 2023;31(3):861–82. 35234976 10.1007/s00167-022-06914-7

[55] Hinterwimmer F, Lazic I, Langer S, Suren C, Charitou F, Hirschmann MT, Matziolis G, Seidl F, Pohlig F, Rueckert D, et al. Prediction of complications and surgery duration in primary TKA with high accuracy using machine learning with arthroplasty-specific data. Knee Surg Sports Traumatol Arthrosc. 2023;31(4):1323–33. 35394135 10.1007/s00167-022-06957-w PMC10050062

[56] Longo UG, Maffulli N, Denaro V. Minimally invasive total knee arthroplasty. N Engl J Med. 2009;361(6):633–4 author reply 634. 19662676 10.1056/NEJMc091111

[57] Lambrechts A, Wirix-Speetjens R, Maes F, Van Huffel S. Artificial intelligence based patient-specific preoperative planning algorithm for total knee arthroplasty. Front Robot AI. 2022;9:840282. 35350703 10.3389/frobt.2022.840282 PMC8957999

[58] Berton A, Longo UG, Candela V, Fioravanti S, Giannone L, Arcangeli V, Alciati V, Berton C, Facchinetti G, Marchetti A, et al. Virtual Reality, Augmented Reality, Gamification, and Telerehabilitation: Psychological Impact on Orthopedic Patients' Rehabilitation. J Clin Med. 2020;9(8). 10.3390/jcm9082567 PMC7465609 32784745

[59] Goplen CM, Kang SH, Randell JR, et al. Effect of preoperative long-term opioid therapy on patient outcomes after total knee arthroplasty: an analysis of multicentre population-based administrative data. Can J Surg. 2021;64(2):E135–43. 10.1503/cjs.007319. 33666382 10.1503/cjs.007319 PMC8064248

[60] Longo UG, Ciuffreda M, Mannering N, D’Andrea V, Cimmino M, Denaro V. Patellar resurfacing in total knee arthroplasty: systematic review and meta-analysis. J Arthroplasty. 2018;33(2):620–32. 29032861 10.1016/j.arth.2017.08.041

[61] Bravi M, Longo UG, Laurito A, Greco A, Marino M, Maselli M, Sterzi S, Santacaterina F. Supervised versus unsupervised rehabilitation following total knee arthroplasty: a systematic review and meta-analysis. Knee. 2023;40:71–89. 36410253 10.1016/j.knee.2022.11.013

[62] Longo UG, Silva S, Perdisa F, Salvatore G, Filardo G, Berton A, Piergentili I, Denaro V. Gender related results in total knee arthroplasty: a 15-year evaluation of the Italian population. Arch Orthop Trauma Surg. 2023;143(3):1185–92. 34665302 10.1007/s00402-021-04222-2

[63] Longo UG, Loppini M, Trovato U, Rizzello G, Maffulli N, Denaro V. No difference between unicompartmental versus total knee arthroplasty for the management of medial osteoarthtritis of the knee in the same patient: a systematic review and pooling data analysis. Br Med Bull. 2015;114(1):65–73. 25743408 10.1093/bmb/ldv009

[64] Longo UG, Ciuffreda M, Mannering N, D’Andrea V, Locher J, Salvatore G, Denaro V. Outcomes of posterior-stabilized compared with cruciate-retaining total knee arthroplasty. J Knee Surg. 2018;31(4):321–40. 28666292 10.1055/s-0037-1603902

[65] Stelfox HT, Gandhi TK, Orav EJ, Gustafson ML. The relation of patient satisfaction with complaints against physicians and malpractice lawsuits. Am J Med. 2005;118(10):1126–33. 16194644 10.1016/j.amjmed.2005.01.060

